# Editorial: Physiological Adaptations to Swimming in Fish

**DOI:** 10.3389/fphys.2017.00059

**Published:** 2017-02-07

**Authors:** Josep V. Planas, Arjan P. Palstra, Leonardo J. Magnoni

**Affiliations:** ^1^Departament de Fisiologia i Immunologia, Facultat de Biologia, Universitat de BarcelonaBarcelona, Spain; ^2^Animal Breeding and Genomics Centre, Wageningen Livestock Research, Wageningen University and ResearchWageningen, Netherlands; ^3^Centro Interdisciplinar de Investigacoes Marinhas e MedioambientalesPorto, Portugal

**Keywords:** fish, performance, swimming exercise, growth, swimming economy

Swimming is an integral part of the life history of many fish species as is intimately linked with their ability to express feeding and predator avoidance behaviors, habitat selection and environmental preferences, social and reproductive behaviors as well as migratory behaviors (Videler, 1993; Palstra and Planas, 2011). Therefore, swimming is an important determinant factor of fitness in a true Darwinian sense and, not surprisingly, swimming performance has been often used as a measure of physiological fitness in fish (Hammer, 2005). In the face of growing changes in the aquatic environment due to global warming and other anthropogenic influences (e.g., hydropower plants and pumping stations, pollution, destruction of essential habitats, etc.), swimming performance can become a relevant proxy for the level of fitness in our evaluation of organismal responses to environmental perturbations in wild fish populations. Changes in the locomotory capabilities of fish due to alterations in swimming performance can have important consequences at the population level in terms of individual dispersal and species abundance, reproductive success and genetic structure of the fish populations, as shown in other vertebrate groups (Hillman et al., 2014). Reduced activity levels due to swimming in captivity can also decrease their physiological fitness status or condition as it is known to occur in aquaculture, when fish cannot display their normal swimming behavior due to confinement under high densities or to insufficient water flows to induce swimming, leading to decreased fitness (both physical and reproductive), growth, survival and muscle quality, depending on the swimming characteristics of the species (Palstra and Planas, 2013). An extensive body of literature supports the notion that swimming, through the ensuing muscle contraction and activation of the cardiovascular system, affects the physiology of the fish through adaptive mechanisms that are recently beginning to be uncovered (Palstra and Planas, 2013; Rodnick and Planas, 2016). Further research efforts in this area should inform the scientific community and the public on the ability of wild fish populations to cope with environmental change and on the benefits of induced swimming for improved aquaculture production and fish welfare.

The main aim of this Research Topic is to showcase some of the current studies designed to improve our understanding of the physiological energetic and metabolic requirements of swimming and of the adaptive responses to swimming in fish. A total of 9 articles are presented, covering topics related to swimming performance in wild and aquaculture-relevant species. The first three articles report on swimming capacity and energetics in different fish species. Tudorache et al. provide a first comparison of swimming capacity and energetic profiles between European eel (*Anguilla Anguilla)* and New Zealand short-finned eel (*A. australis)*, two species that differ in the extent of their migration to their potential spawning sites. Their results indicate that European eels have higher swimming capacity but that the two species show similar energetic profiles during swimming. Killen et al. investigate the trade-offs associated with predator avoidance and energy balance across a range of temperatures in the golden gray mullet (*Liza aurata*). Their results show that fish that respond first to danger and that show higher escape performance are characterized by higher aerobic scope (AS) and longer recovery rates independently of temperature, suggesting that the energetic demands of vigilance, possibly due to higher brain activity, prolong physiological recovery after anaerobic exercise. Svendsen et al. report on the metabolic costs of swimming in relation to the trade-off between aerobic and anaerobic traits in gilthead sea bream (*Sparus aurata*). Their results show that fish with high maximum sustained swimming speed (U_sus_) also exhibit high optimum swimming speed (U_opt_) and low minimum cost of transport (COT), suggesting that high U_sus_ and minimum COT may be optimized concurrently and that burst swimming is associated with anaerobic metabolism and a substantial metabolic cost. Moreover, four other articles describe the effects of swimming on growth and performance. Skov et al. report on the effects of swimming under dietary restriction on growth in rainbow trout (*Oncorhynchus mykiss*). Their results show that trout swimming under sustained conditions and dietary restriction experience increased relative metabolic expenditure resulting in decreased specific growth rate and feed conversion ratio but without an increase in the use of protein as fuel. In contrast, Palstra et al. report that yellowtail kingfish (*Seriola lalandi*) swimming at their established U_opt_ show increased growth performance, feeding efficiency and cardiac output, in a clear example of a species benefiting from the physiological effects of swimming. Anttila et al. report on the relationship between swimming performance and cardiorespiratory performance and morphology in the context of thermal tolerance in Atlantic salmon (*Salmo salar*). Their results show that good swimmers had improved cardiorespiratory features including thicker cardiac compact layer and taller gill secondary lamella but similar thermal tolerance and that these benefits of swimming persisted after 8 months coupled with an increase in growth rate. In the last article on growth, Khan et al. report on the swimming conditions that promote optimal growth rates and the possible influence of AS in juvenile hapuku (*Polyprion oxygeneios*). Their results indicate that juvenile hapuku show a limited growth response to swimming and that AS does not appear to limit exercise-induced growth in this species. In an attempt at describing the mechanisms responsible for the physiological benefits of swimming on skeletal muscle, the paper by Morash et al. provides information on the metabolic adaptive responses to sustained swimming in rainbow trout. Their results show that sustained swimming elicits distinct changes in red and white skeletal muscle in terms of activity and expression of metabolic markers, suggestive of phenotypic plasticity in skeletal muscle. Finally, Pelster reports on the physiological function of the swimbladder of the European eel and its role in swimming during migration. In this article, the physiological role of the swimbladder, its energetic requirements and nematode infections are discussed in the light of the European eel's reproductive migration requirements. We hope that this Research Topic will be of interest to researchers in and outside the field and will stimulate them to consider including a swimming physiological perspective in their studies.

## Author contributions

JP, AP, and LM all contributed equally to write this editorial.

### Conflict of interest statement

The authors declare that the research was conducted in the absence of any commercial or financial relationships that could be construed as a potential conflict of interest.
